# In Situ Ultrasonic Characterization of Hydrogen Damage Evolution in X80 Pipeline Steel

**DOI:** 10.3390/ma17235891

**Published:** 2024-12-01

**Authors:** Bing Chen, Feifei Qiu, Li Xia, Lintao Xu, Junjun Jin, Guoqing Gou

**Affiliations:** 1 School of Materials Science & Engineering, Key Laboratory of Advanced Technologies of Materials (Ministry of Education), Southwest Jiaotong University, Chengdu 610031, China; 2 Zhejiang Academy of Special Equipment Science, Hangzhou 310009, China

**Keywords:** in situ ultrasonic characterization, hydrogen damage evolution, hydrogen-induced crack, X80 pipeline steel

## Abstract

A nondestructive evaluation of the hydrogen damage of materials in a hydrogen environment is important for monitoring the running conditions of various pieces of equipment. In this work, a new thermostatic electrolytic hydrogenation in situ ultrasonic test system (In Situ TEH-UT) was developed. The system operates by combining cross-correlation delay estimation and frequency domain amplitude estimation and hence improves measurement accuracy with respect to ultrasonic propagation time and amplitude, allowing in situ ultrasonic evaluation of the hydrogen-charging process in X80 pipeline steel. The experimental results show that under a 30 mA/cm^2^ hydrogen-charging current, the hydrogen saturation time of X80 pipeline steel is 800 min. Between 0 and 800 min, the attenuation coefficient and amplitude attenuation both demonstrate a strong linear relationship with the hydrogen-charging time. After 800 min, the attenuation coefficient and amplitude attenuation do not change further, while the attenuation coefficient fluctuates greatly. Through the characterization of the microstructures of the materials analyzed, it was found that hydrogen-induced cracks (HICs) constituted the main reason for the change in the ultrasonic parameters, and the mechanism behind the hydrogen-induced damage layer (HIDL) was determined. This study provides reference significance for clarifying the change mechanism of ultrasonic parameters under hydrogen damage conditions and determining the extent of hydrogen damage using an ultrasonic technique.

## 1. Introduction

With the increasing demand for oil and gas, pipeline transportation has become the first choice for oil and gas delivery due to its low cost, high efficiency, safety, and minimal environmental impact [1,2]. However, among these multiple aspects, the safety of pipeline transportation is affected by many factors; specifically, damage and failure caused by the introduction of hydrogen atoms in pipeline steels are found more and more frequently [3,4]. Currently, X80 pipeline steel is widely used in pipeline transportation because of its high strength, good ductility, and, in particular, its satisfactory walkability [5]. However, it has also been realized that in a hydrogen atmosphere (petroleum, natural gas, coal gas, cathodic protection, etc.), X80 steel is prone to interacting with hydrogen atoms, resulting in corresponding hydrogen damage and failure [6,7]. This makes it essential to be able to carry out non-destructive testing and monitoring of the degree of hydrogen damage in this type of steel since the extraction and transportation of oil and gas generally occur in remote regions far from human society.

As is well known, destructive testing methods were often used to evaluate hydrogen damage in materials in the early days, including methods like metallographic analysis [8], the slow strain rate tension test (SSRT) [9], toughness tests [10], fracture surface analysis [11], etc. Zhou et al. studied the effect of internal hydrogen and surface hydrogen absorption on the hydrogen embrittlement of X80 pipeline steel via a tensile test before and after annealing and a hydrogen permeation test with different strain rates [12]. However, these destructive tests are not convenient for online hydrogen damage detection for in-service equipment. Non-destructive testing (NDT) has thus become an important research topic with regard to evaluating the hydrogen damage inflicted on materials [13]. The NDT approaches generally include eddy current [14], acoustic emission [15], ultrasonic and neutron diffraction [16], etc. Kruger applied non-destructive testing technology and ultrasonic spectrum analysis to steel samples to detect micro-cracks caused by hydrogen in an H_2_S environment and discovered that the second-order moments showed greater variability for hydrogen-attacked materials than for non-attacked ones [17].

As a mainstream nondestructive testing technology, ultrasonic methods can indirectly reflect the microstructural characteristics of different materials and are widely used in the fields of material damage detection and material structure integrity assessment [18,19,20]. In the field of hydrogen damage detection, researchers usually combine acoustic parameters such as ultrasonic speed, attenuation, and various anisotropic coefficients to construct hydrogen damage evaluation models and evaluate the degrees of hydrogen damage in materials. For example, Ye et al. employed surface waves to characterize early hydrogen damage in AISI 304 austenitic stainless steel. Their results showed the high-frequency surface wave velocity would change with hydrogen-induced martensitic transformation when increasing the hydrogen-charging time [21]. Frolova et al. detected the local hydrogen damage zone of rolled steel, along with the acoustic anisotropy magnitude, based on the polarization S-wave probe. They found that the acoustic anisotropy magnitude had a good correlation with the concentration of hydrogen dissolved, and the correlation coefficients of several samples amounted to 0.95 [22]. However, these studies used parallel samples for offline detection. The number of samples that can be obtained using this method is limited, and real-time ultrasonic information during the infliction of hydrogen damage cannot be obtained. For example, monitoring hydrogen damage in hydrogen transport pipelines is an important means of ensuring the safe operation of these pipelines. However, in this type of detection, the pipeline itself cannot be damaged, so damaged offline samples are not applicable, and only non-destructive testing can be used.

In this work, we report a new type of thermostatic electrolytic hydrogenation in situ ultrasonic test system (In Situ TEH-UT). This system was developed to realize the acquisition of ultrasonic signals in the process of continuous hydrogen charging in X80 pipeline steel. The ultrasonic characteristic parameters under different hydrogen-charging times were obtained by using the cross-correlation delay estimation algorithm and frequency domain amplitude estimation algorithm. By means of the multi-sample method and microscopic characterization, the mechanism behind the ultrasonic detection of hydrogen damage in X80 pipeline steel was elucidated, and with the use of this new system, a hydrogen damage detection and prediction model was established.

## 2. Experimental Section

### 2.1. Materials

The X80 pipeline steel employed was supplied by Pipe-China, and its nominal composition is shown in Table 1. The X80 pipeline steel was cut into rectangular samples measuring 30 mm × 20 mm × 8 mm via the wire-cutting method, polished with sandpaper to 800# in a step-by-step fashion, and then fully polished.

### 2.2. Equipment

**Figure 1:**
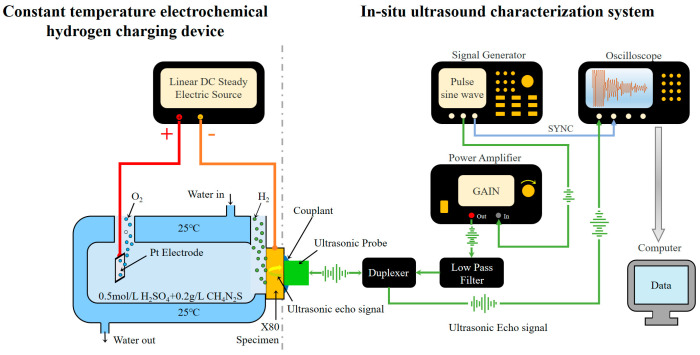
Diagram of In Situ TEH-UT system.

In Situ TEH-UT consists of a thermostatic electrolytic hydrogenation device and an in situ ultrasonic test system, as shown in Figure 1.

#### 2.2.1. Electrochemical Hydrogen Charging

The thermostatic electrolytic hydrogenation device uses a linear DC steady electric source (IT6834, Itech, China), and 0.5 mol/L of H_2_SO_4_ is used as the electrolyte solution for electrochemical hydrogen pre-charging, in which 0.2 g/L of thioureas was added as catalyst. Pt electrode was used as the anode of the hydrogen-charging system, and the specimen was connected to the cathode; the current density for hydrogen pre-charging was 30 mA/cm^2^. A liquid thermostatic bath (HLC-2005E, Huxi, China) was used to circulate cooling water in the outer layer of the double-layered electrolytic cell to ensure the temperature during the hydrogen-charging process remained 25 °C.

#### 2.2.2. Ultrasonic Measurements and Signal Processing

The in situ ultrasonic test system consists of the following parts: a signal generator (AFG3252, Tektronix, America), a power amplifier (AG1016, T&C, America), an oscilloscope (SDS2074X Plus, Siglent, China) to demonstrate the needed signals on a real-time basis, a low-pass filter, a duplexer (for isolating the transmitting and receiving signals in the system), an ultrasonic transducer, and a computer for processing the data collected. The process of hydrogen charging is briefly described as follows. The signal generator generates a burst signal with a center frequency of 2.5 MHz, which is amplified by the power amplifier and filtered by the low-pass filter. An ultrasonic transducer with a fixed center frequency is selected as the transmitting and receiving sensor. The sensor uses lithium grease coupled with the sample and keeps the sensor and the hydrogen-charging surface on the same central axis. After the received signal is recorded and averaged via the oscilloscope, it is transmitted to and processed by the computer, and the ultrasonic parameters of the signal can then be obtained.

**Figure 2:**
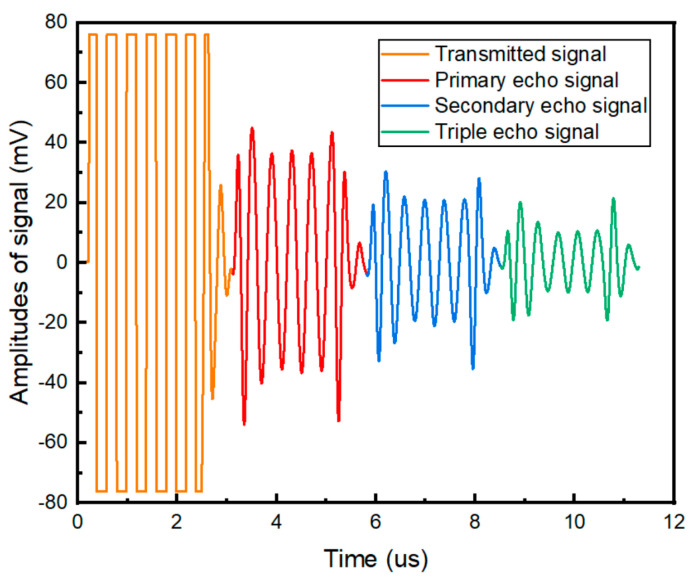
Example of transmitted and received time domain signals.

Figure 2 shows an example of echo time domain signal. In order to ensure that the echo signals do not overlap, the exact number of cycles of the signal burst is set as the maximum number of cycles that can fit within the thickness of the specimen. This eliminates any possible spurious higher harmonics generated by the interference of the incident and reflected wavefronts, as well as the effects of boundary conditions. In this work, the number of cycles was determined to be 7.

Ultrasonic velocity *V* can be expressed as *V* = *2d*/Δ*t*, where *d* is the sample thickness and Δ*t* is the time difference between adjacent echoes. The peak time difference between two echo signals (Find–Peak) is often used to calculate Δ*t*, but the echo signal is weak in the later stage of damage, and hence environmental noise has a great influence on time estimation in this stage. In order to minimize the effect of noise, we employed the average noise reduction to suppress noise energy, and cross-correlation time delay estimation (NCC-TDE) was used to estimate Δ*t*. NCC-TDE is a method in which the cross-correlation function *R*(*x*,*y*) is used to judge the similarity of two waveforms and the position of the *R*(*x*,*y*) peak is determined to estimate the time delay. *R*(*x*,*y*) can be expressed as

R(x,y)=IDFT{DFT{x}*∘DFT{y}}

where *x* and *y* are two sets of signals to be estimated, *DFT* is a discrete Fourier transform, *IDFT* is a discrete Fourier inverse transform, the symbol “∘” denotes the multiplication of corresponding terms of a vector in the same dimension, and the symbol “*” denotes conjugation of complex signals. The implementation process for NCC-TDE is as follows:

① Via Fourier transform, the primary echo signal *x*(*n*), the secondary echo signal *y*(*n*), and the spectrum signals *Zx*(*n*) and *Zy*(*n*) are obtained;
② The conjugate of *Zx*(*n*) is determined, yielding *Zx*(*n*)*;
③ The spectrum of the cross-correlation function *Z*(*R*(*x*,*y*)) is obtained by multiplying *Zx*(*n*)* and *Zy*(*n*) elements;
④ The inverse Fourier transform of *Z*(*R*(*x*,*y*)) is the cross-correlation function *R*(*x*,*y*);
⑤ The peak position of *R*(*x*,*y*) is the time difference between adjacent echoes Δ*t*.

**Figure 3:**
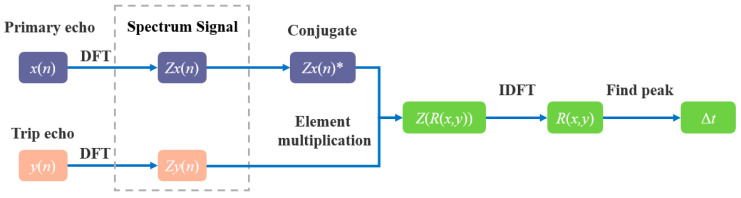
Flowchart of cross-correlation time delay estimation.

A flowchart of NCC-TDE is shown in Figure 3.

**Figure 4:**
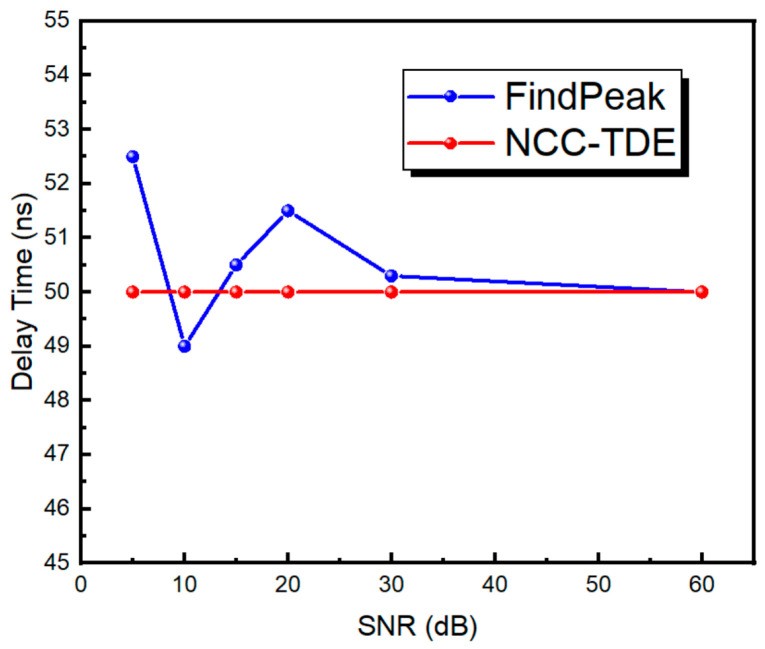
NCC-TDE and Find–Peak anti-noise effect comparison.

Figure 4 shows the delay estimation accuracy of NCC-TDE with a fixed delay of 50 ns and different signal-to-noise ratios (SNRs). Under the condition of a high SNR, Find–Peak and NCC-TDE have the same effect. However, with the decrease in SNR, the waveform signal deteriorates, and Find–Peak calculation results become unstable. However, under the condition of a low SNR, the cross-correlation delay algorithm can reduce noise to a certain extent; thus, the calculation results are stable. Based on the above discussion, all subsequent delay estimation algorithms adopted in this work correspond to cross-correlation delay estimation.

**Figure 5:**
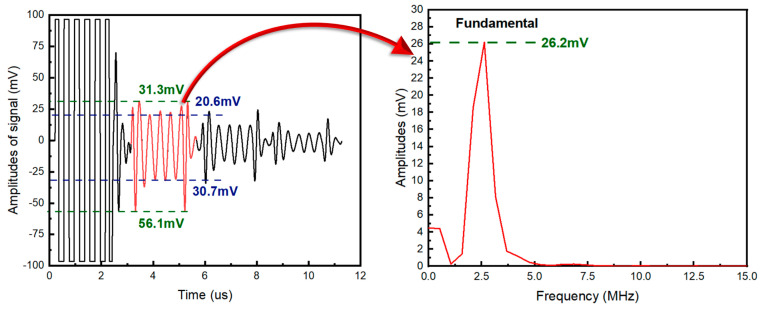
Time and frequency domain signals of uncharged hydrogen samples.

The attenuation coefficient can be calculated using the difference in the ultrasonic echo amplitude. The calculation formula is given below:

$$\alpha =20\frac{\log_{10}(B_{n}/B_{m})}{2(m-n)d}$$

where *B_n_* and *B_m_* are the amplitudes of the echoes *n* and *m* times, respectively. In this work, the first and second echoes were selected. Similarly, amplitude attenuation can be expressed as

$$\beta =20\log_{10}(B_{n}^{1}/B_{n}^{j}),$$

where $B_{n}^{1}$ and $B_{n}^{j}$ represent the amplitude of the 1st and jth measured echo signal. Due to the presence of noise and high-order harmonic signals generated by nonlinear ultrasonic effects in ultrasonic time-domain signals, there is a certain error if the maximum echo value is utilized to directly calculate the attenuation coefficient. Therefore, the frequency domain analysis method was adopted in this work. The ultrasonic echo time-domain signals were obtained using Fourier transform, and the fundamental frequency amplitude was used as the echo amplitude to calculate the attenuation coefficient and amplitude attenuation degree. Figure 5 shows the time delay signal of a group of echo signals with a base frequency of 2.5 MHz and the signal in the frequency domain of a primary echo. It can be seen that the amplitude of the time delay signal of the primary echo fluctuates in different positions due to the influence of high-order harmonics and noise, while the amplitude of the fundamental frequency in the frequency domain is relatively stable.

## 3. Results and Discussion

### 3.1. Ultrasonic Behavior as Damage Is Inflicted

A rectangular sample measuring 30 mm × 20 mm × 8 mm was charged with hydrogen at a hydrogen-charging current density of 30 mA/cm^2^, and ultrasonic signals were collected continuously at an interval of 1 min. The cross-correlation delay estimation algorithm and frequency domain amplitude estimation algorithm were used to calculate the echo sound velocity *V*, attenuation coefficient *α*, and amplitude attenuation degree *β*.

**Figure 6:**
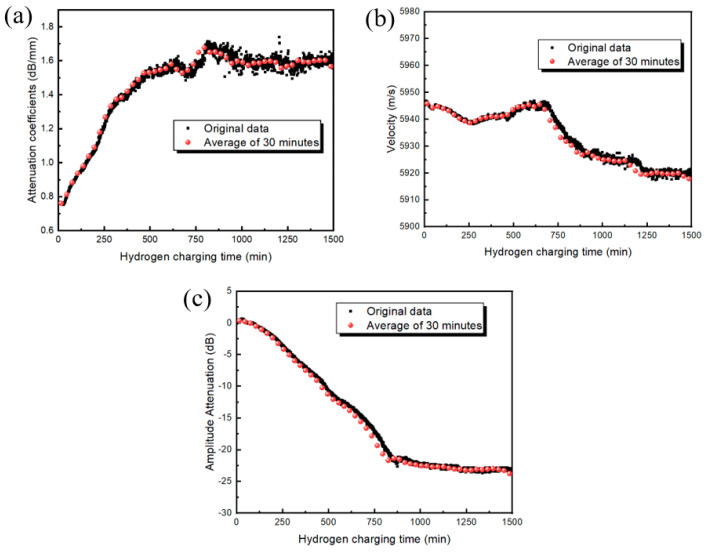
Attenuation coefficient, amplitude attenuation, and sound velocity of echo signal: (**a**) attenuation coefficient *α*; (**b**) velocity; and (**c**) amplitude attenuation *β.*

The attenuation coefficient and amplitude attenuation of an ultrasonic echo can be used to measure the degree of hydrogen damage inflicted on X80 pipeline steel, as shown in Figure 6. The results show that as the cumulative attenuation coefficient of hydrogen damage increases, the amplitude attenuation decreases in the late stage of hydrogen damage (after 800 min). The variation rate for the attenuation coefficient and amplitude attenuation weakens and gradually tends to a constant value, at which time the attenuation coefficient exhibits increased fluctuation. The ultrasonic sound velocity does not appear to be sensitive to the degree of hydrogen damage in X80 pipeline steel. The maximum propagation velocity difference is less than 45 m/s, while the linearity between the velocity and charging time is poor, so the velocity response should not be selected for hydrogen damage detection.

**Figure 7:**
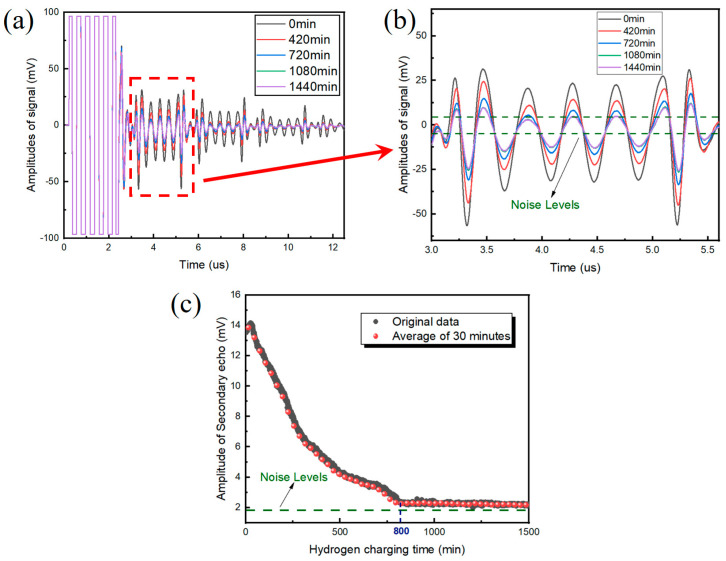
Ultrasonic waveforms with respect to different hydrogen-charging times: (**a**) overall signal; (**b**) secondary echo signal; and (**c**) amplitude of secondary echo signal.

Figure 7 depicts the ultrasonic waveform under different hydrogen-charging times. It can be seen that after 800 min of hydrogen charging, the amplitude of the secondary loop attenuation is almost consistent with the noise level, resulting in an inaccurate calculation of the attenuation coefficient as well as fluctuation in the attenuation coefficient. In contrast, the amplitude attenuation fluctuates less in both the early and late stages of hydrogen damage. Based on these observations, it can be concluded that amplitude attenuation is more suitable for hydrogen damage detection than the attenuation coefficient.

### 3.2. Microstructural Changes as Damage Is Inflicted

According to Figure 7c, the amplitude of the ultrasonic signal was consistent with the noise level after 800 min, so the change in ultrasonic parameters caused by hydrogen was mainly concentrated below 800 min. In order to explore the mechanism of the ultrasonic detection of hydrogen damage, the characteristic time period (60 min, 210 min, 420 min, 720 min, 1080 min, and 1440 min) was selected, and the hydrogen-charging current was set to 30 mA/cm^2^ for the hydrogen charging of several samples. After hydrogen charging for each time period, the surfaces of the samples were immediately washed with acetone solution to prevent corrosion and lightly polished with a polishing cloth. Then, the surface states of the samples were observed with a microscope (JYWD-SZ61, Olympus, America).

**Figure 8:**
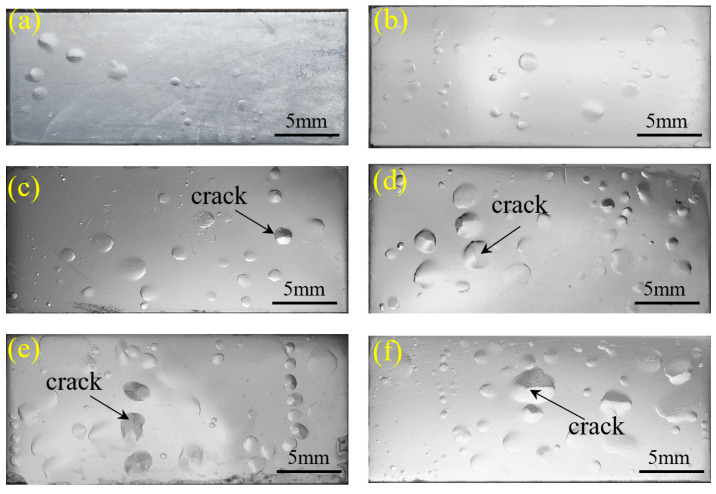
Hydrogen blisters in X80 steel samples subjected to hydrogen charging for 60 min (**a**), 210 min (**b**), 420 min (**c**), 720 min (**d**), 1080 min (**e**), and 1440 min (**f**).

The surface topography of hydrogen bubbles in X80 steel is shown in Figure 8, where (a) and (b) show the surface morphologies of hydrogen bubbles after 60 min and 210 min of hydrogen charging, respectively. At this time, the number of hydrogen bubbles kept increasing and no apparent cracks could be seen. When the hydrogen-charging time was greater than 420 min, the amount of hydrogen blistering continues to increase, and some of the bubbles burst and form cracks, as shown in (c)–(f).

**Figure 9:**
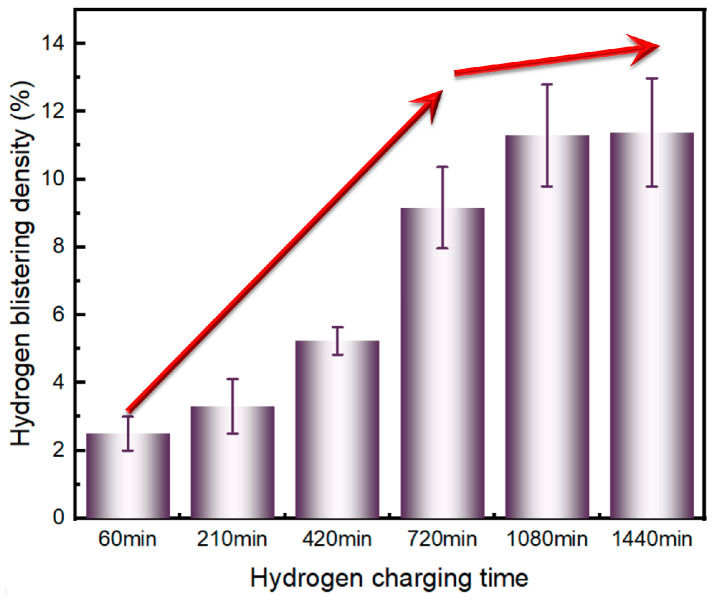
Hydrogen blistering density vs. hydrogen-charging time on the surface of X80 steel.

The hydrogen-blistering density of X80 at different charging times is shown in Figure 9. With the extension of the hydrogen-charging time, the density of hydrogen bubbles gradually increased. For the charging period from 0 to of 720 min, the growth rate for hydrogen blistering density was found to be 0.0127 min^−1^; between 720 and 1080 min, the growth rate of hydrogen blistering density slowed down significantly and was found to have only decreased to 0.0059 min^−1^. After 1080 min, the hydrogen blistering density was almost unchanged, a phenomenon caused by the saturation of the hydrogen concentration. This trend is consistent with the conclusions given in Figure 6.

**Figure 10:**
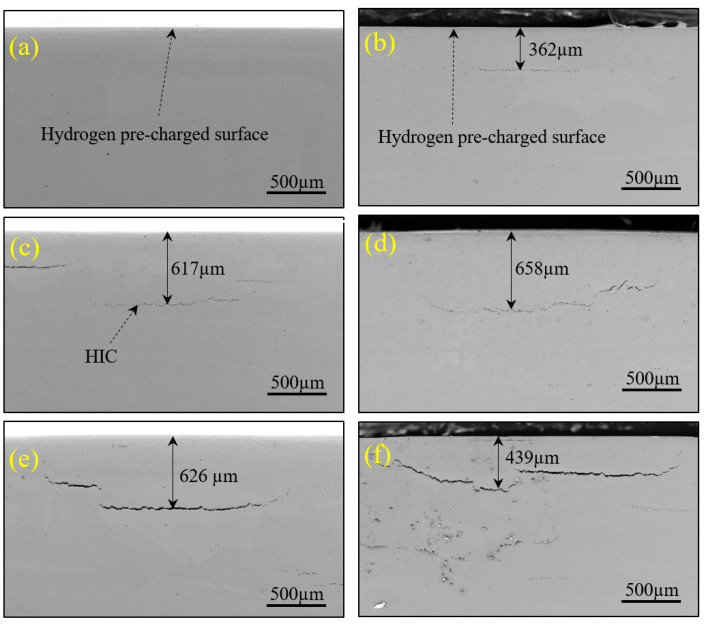
SEM images of HICs in X80 steel samples at hydrogen pre-charging times of 60 min (**a**), 210 min (**b**), 420 min (**c**), 720 min (**d**), 1080 min (**e**), and 1440 min (**f**).

The samples with bubbles on the surface that were electrochemically hydrogen-charged were cut along the cross section of the bubbles so as to detect and observe HICs using a scanning electron microscope (Gemini 300, Zeiss, Germany). Indeed, stepped cracks with similar surface blistering sizes were found beneath the surfaces on which hydrogen blistering took place. The majority of these cracks were all parallel to the surface, but some were perpendicular to the surface, as shown in Figure 10. Furthermore, crack size was found to increase with the increase in the hydrogen-charging time. It was also found that the maximum depth of the cracks detected is less than 700 µm, a result that generally conforms to the results in Ref. [23].

After hydrogen atoms penetrated the metal, they concentrated mainly on its hydrogen-pre-charged surface. These broad-sense defects at or near the sample surface are hence the initiation sites of hydrogen cracks. With the increase in hydrogen-charging time, the hydrogen content in the sample also increased. When the hydrogen pressure inside the hydrogen bubbles increased and exceeded the material’s yield strength, blistering occurred, leading to HIC formation.

Based on the above interpretation of the hydrogen damage behavior and the change pattern of the ultrasonic parameters, the mechanism of the hydrogen damage in X80 pipeline steel detected via ultrasound can be explained as follows. At the beginning of hydrogen pre-charging, hydrogen atoms enter the metal. They concentrate mainly in the hydrogen-pre-charged surface of the metal and induce hydrogen blistering, causing changes in the surface morphology of the hydrogen-charged steel, resulting in a gradual changes in ultrasonic reflection from total reflection to diffused reflection, leading to the weakening of the intensity of the as-received echo signal. With the increase in hydrogen-charging time, the hydrogen pressure will exceed the yield strength of the base metal, and cracks will form on the surface of the material. The size of the HICs keeps increasing as the charging time elapses, so the ultrasonic wave is unable to penetrate the cracks. Thus, the echo intensity is gradually weakened. When the hydrogen-charging time exceeds 800 min, the hydrogen concentration of the sample reaches saturation. This will slow down and eventually stop the growth of the HICs, leading to the stabilization of the echo intensity. In this work, such a mechanism is designated as the formation of a hydrogen-induced damage layer (HIDL).

The phenomenon of HIDL formation indicates that HICs constitute the main factor altering the ultrasonic parameters, and the mechanism tended to appear on the shallow surface of the hydrogen-charged sample. Therefore, if the hydrogen-charged sample surface, even if charged for a long time (after 800 min), is polished layer by layer, eventually, the ultrasonic parameters of this sample should return to their initial state without hydrogen charging when the hydrogen-induced crack layer has been completely polished.

**Figure 11:**
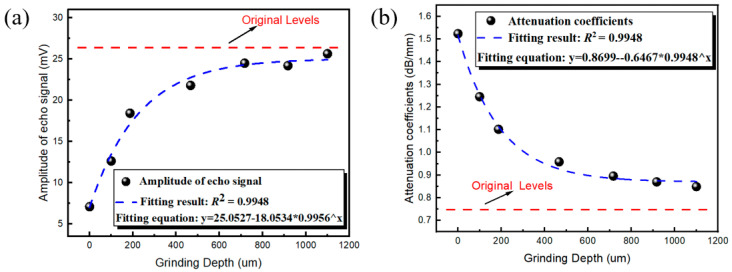
The amplitude (**a**) and attenuation coefficient (**b**) at different grinding depths.

Figure 11 shows the attenuation coefficient and amplitude corresponding to different grinding depths of the sample following hydrogen charging for 1500 min. It can be seen that with the increase in grinding depth, the amplitude of the return wave gradually increases and the attenuation coefficient gradually decreases; when the grinding depth exceeds 700 µm, the attenuation coefficient and amplitude tend to be stable and close to the level of the sample that was not subjected to hydrogen charging. This also verifies the formation mechanism of the HIDLs.

It should be noted that the hydrogen atoms entering the metal exist in the interstitial space of the lattice. Under stress, the hydrogen atoms will spread to the stress concentration area at the front of the defect or crack, hindering the dislocation movement in the area, resulting in local work hardening and improving the resistance of the metal to plastic deformation. The effect of stress will further aggravate the hydrogen damage degree of the material. Therefore, it is necessary to further study hydrogen damage behavior under stress and understand the relationship between hydrogen damage under stress and ultrasonic characteristics. In Situ TEH-UT uses water as a constant-temperature medium, so the monitoring temperature is near room temperature; it is not suitable for pipelines with higher service temperatures (100 °C and above). At the same time, In Situ TEH-UT can only be used to evaluate hydrogen damage, and pipeline life is affected by stress, corrosion, and other aspects. In Situ TEH-UT is not yet suitable for practical multifactorial environments.

## 4. Conclusions

In summary, we developed a new system, In Situ TEH-UT, in this work for in situ ultrasonic testing in the process of the hydrogen charging of X80 pipeline steel, and the microscopic principles of the mechanism of the hydrogen damage in X80 pipeline steel detected via ultrasound were analyzed based on SEM data. This study provides reference significance for clarifying the change mechanism of ultrasonic parameters under hydrogen damage and the determination of hydrogen damage using the ultrasonic technique. Several main conclusions have been drawn:

(1) The attenuation coefficient and amplitude attenuation of the ultrasonic echo signal were highly sensitive to the hydrogen damage behavior of the X80 pipeline steel, especially when the amplitude attenuation reached 800 min, at which point its value fluctuated less and it was more suitable for hydrogen damage detection. And these parameters have a good linear correlation with hydrogen-charging time.
(2) With the increase in hydrogen-charging time, the hydrogen-blistering density and HIC size increased. After 800 min, both of them reached a stable state. HICs mainly appeared within 700 µm of the hydrogen-charging surface.
(3) We first reported the mechanism of HIDL formation: we consider the change in ultrasonic parameters to be mainly related to the hydrogen cracks on the hydrogen-filled surface. The results of the layer-by-layer grinding test show that the attenuation coefficient and amplitude do not change when the grinding depth exceeds 700 µm, which is close to the original sample level. This work makes it possible to apply ultrasonic test technology to identify and predict the hydrogen damage of X80 pipeline steel and ensure the structural integrity of steel.

## Figures and Tables

**Table 1:** Chemical composition of X80 pipeline steel (wt.%).

C	Si	Mn	P	S	Al	V
0.0699	0.177	1.642	≤0.085	≤0.0018	0.268	0.0254
Nb	Ti	Cr	Mo	Ni	Cu	H
0.0495	0.0124	0.085	0.195	0.255	0.14	≤2.5 ppm

## Data Availability

The original contributions presented in this study are included in the article. Further inquiries can be directed to the corresponding author.
